# Feasibility study of Internet video-based speech-language activity for outpatients with primary progressive aphasia

**DOI:** 10.1371/journal.pone.0288468

**Published:** 2023-07-13

**Authors:** Shinji Higashi, Yuko Koshibe, Takehiro Miyazaki, Ryohei Watanabe, Hanako Nakanome, Takeshi Inoue, Takashi Asada, Tetsuaki Arai

**Affiliations:** 1 Department of Psychiatry, Ibaraki Medical Center, Tokyo Medical University, Ibaraki, Japan; 2 Memory Clinic Ochanomizu, Bunkyo-ku, Tokyo, Japan; 3 Division of Clinical Medicine, Department of Psychiatry, Faculty of Medicine, University of Tsukuba, Tsukuba, Ibaraki, Japan; 4 Department of Psychiatry, Tokyo Medical University, Shinjuku-ku, Tokyo, Japan; Kazawa University Graduate School of Medical Sciences, JAPAN

## Abstract

**Background:**

Primary progressive aphasia is a clinical dementia syndrome secondary to neurodegenerative disease characterized by language-related difficulties. Currently, there is no effective treatment for language impairment in primary progressive aphasia. In the present study, we investigated the feasibility of Internet video-based speech-language activities for this condition.

**Methods:**

Twenty-three people with primary progressive aphasia (pwPPA) participated in the study and were provided with twelve speech-language activity videos on a dedicated website, with three sessions per week. The group that chose to continue with participation after three months of intervention received Internet activities for one year. Cognitive domains associated with persistence, treatment motivation, and video difficulty settings were statistically analyzed.

**Results:**

After three months, 17 out of 23 participants opted to continue with the activities. The ability to follow oral commands which was measured pre intervention was higher in the group that continued compared with those participants who discontinued activity. The scores of two Standard Language Test of Aphasia subtests, sentence repetition and narrative writing–associated with the ability to comprehend and produce sentence structure–were highly correlated with motivation, interest and concentration in activity. Participants with different levels of primary progressive aphasia progression could participate in the same video-based activities when high-frequency words were used in the video.

**Conclusions:**

Internet video-based speech-language activity at home has potential as a useful tool for future primary progressive aphasia treatment because it provides a cost-effective approach to intensive intervention and overcomes barriers associated with traditional therapy approaches.

## 1. Introduction

Recent clinicopathological and genetic studies have revealed the details of brain changes in neurodegenerative diseases. Alzheimer’s disease (AD) and dementia with Lewy bodies (DLB) are neuropathologically defined by the pathological structures and their component proteins that appear in the patient’s brain. Frontotemporal lobar degeneration (FTLD), in contrast, is composed of pathologically and genetically distinct diseases based on a disease concept characterized by degenerative regions of the brain. Thus, neurodegenerative diseases are classified according to pathological findings and/or degenerative areas of the brain.

Primary progressive aphasia (PPA), proposed in 2011, is a clinical dementia syndrome secondary to neurodegenerative disease. It is diagnosed according to its clinical manifestations of language-related difficulties and neuroimaging examinations. These gradually progressive impairments affect language production, object nomenclature, syntax, word comprehension, and reading and writing. PPA was first defined by Mesulam et al. in 1982 [1] and clinical diagnostic criteria were later proposed by Gorno-Tempini et al. [2]. Clinical diagnosis of PPA requires that language impairment be the predominant clinical manifestation early in the course of the disease. PPA is classified into three types: semantic variant PPA (svPPA), non-fluent/agrammatic variant PPA (naPPA), and logopenic variant PPA (lvPPA). svPPA pathologically corresponds to FTLD with TDP-43 positive inclusions (FTLD-TDP), whereas more than half of lvPPA cases are associated with AD [3, 4]. naPPA is often associated with FTLD with tau-positive inclusions (FTLD-tau) and can be FTLD-TDP, AD or DLB [3, 4].

Pilot studies of PPA show the effectiveness of non-pharmacological therapies, such as speech-language therapy [5–8]. PPA is classified into several variants that impair naming, sentence production, speech production, and phonological awareness. Speech-language therapy focuses on one or some of these deficits. Previous studies have proposed several therapies shown to be effective for PPA. Speech-language therapy for naming deficit includes lexical retrieval treatment, phonological and/or orthographic treatment, semantic treatment, and a multimodality treatment approach [9], and script training has been reported for agrammatic PPA [8]. Intervention methods may be verbal or written; pictorial stimulation is common, and errorless methods are also employed [10, 11].While many studies have shown statistically significant improvements, maintenance effects vary, and there are many reports of effects declining with treatment discontinuation. Recent reports describe increased efficacy with the use of transcranial direct current stimulation (tDCS) [12, 13], and significant changes in functional connectivity with tDCS and speech-language therapy have been observed by fMRI [14], raising hopes of continued therapeutic efficacy. Most studies have reported face-to-face therapy. However, when frequent face-to-face treatment is difficult because of limited medical resources, remote systems may provide more treatment opportunities and enhance the effectiveness of non-pharmacological interventions. Information technology, including Internet videos, may be particularly effective because paper-based treatment has the disadvantage of eliminating the act of listening and speaking. The COVID-19 pandemic also reduced opportunities for face-to-face treatment and demonstrated technology’s critical role in ensuring speech-language therapy that can be implemented under these circumstances.

Therefore, this study investigated the feasibility of home-based speech-language activities for people with PPA (pwPPA) using online videos. There were concerns about the pwPPA maintaining motivation in activities conducted alone at home, and that online videos uploaded from the Internet could not be customized for each individual regarding difficulty level and video speed. Thus, we conducted an autonomous speech-language activity at home using online internet videos with the same content and speed for PPA with varying severities and symptoms, as well as the cognitive domains that influence the realization and discussed whether the content, speed, or difficulty of the video needs to be adjusted for each person. The goal of this study was not to investigate the effectiveness or efficacy of the intervention, but examine the specific uncertainties associated with conducting a larger study, such as the user acceptability or adherence to the intervention.

## 2. Materials and methods

### 2.1 Participants

Twenty-three right-handed pwPPA, including 14 people with lvPPA, 6 with non-fluent and/or agrammatic variant PPA, and 3 with svPPA, were recruited from the Departments of Psychiatry at the Tokyo Medical University Ibaraki Medical Center, the University of Tsukuba Hospital and the Memory Clinic Ochanomizu between November 2020 and March 2021. The participants took part in a prospective study of speech activities at home using online videos. Clinical diagnosis of PPA was made by one of three medical doctors (SH, TA or TA) based on clinical symptoms, neurological findings, and neuroimaging examinations after the following neuropsychological examinations by the same speech-language pathologists (YK), following the PPA criteria from Gorno-Tempini et al. [2]. The group contained 15 male participants and 8 female participants. Mean (±SD) age at the time of participation was 67.7 (±10.7) years with a range of 51–87 years. Symptom duration (±SD) had a mean of 4.4±2.0 years. Mean (±SD) score for the Mini-Mental State Examination (MMSE) was 18.8±6.2, and Frontal Assessment Battery (FAB) mean score was 11.7 (±3.4). Raven’s colored progressive matrices (RCPM) and the Geriatric depression scale (GDS) had mean scores of 26.9 (±6.6) and 3.9 (±2.0), respectively.

### 2.2 Study design and procedure

This study was a prospective observational study conducted to assess intervention. The study was approved by the Institutional Review Board of the University of Tsukuba (Registration No.R02-182) and Tokyo Medical University (Registration No.T2020-0421). Participants were those who attended one of the three medical facilities as outpatients during the period described above, were diagnosed with PPA at their first visit, and consented to participate. During the study period, the participants continued to attend outpatient clinics once every one to two months, and if they stopped attending, study participation was stopped. This was because it was not possible to collect the answer/questionnaire booklet described below. Patients with dementia who were not diagnosed with PPA were excluded, but the results of neuropsychological examinations were not part of the inclusion criteria. Although the presence of a collaborator to support speech-language activities was not a criterion for inclusion, 18 of the 23 participants had a collaborator. They gave their informed written consent to participate. Speech-language activity took place at home using online videos of approximately 10 minutes duration. Twelve videos were created with different language content to encourage comprehension, reading aloud, dictation and/or vocalization of language components, such as nouns, verbs, sentences and numbers. These language components were changed for each video. The information was presented on the screen with letters, pictures, animations and/or auditory stimuli (Table 1 and S1 and S2 Appendices). Words with high familiarity (>5.5) were used for approximately 90% of the words in the Internet videos based on a Japanese language database [15]. A “nonverbal word” was created with reference to a Sophia analysis of language in aphasia (SALA) by converting some of the mora in a real word into other ones [16]. All online videos were uploaded through Vimeo (https://vimeo.com/jp/), and one of the 12 videos was made available to participants by embedding it on a dedicated Internet homepage. The uploaded videos were replaced with new videos three times a week and were available for viewing during that time, i.e. only one video was embedded on the homepage, which contained both explanatory and homework sections (See S1–S12 Appendices) and was set up with no speed changes. All 12 videos were provided over a 4-week period, making this into one activity course. All of the videos used in this study newly created and uploaded for each session. There was no homework in this study other than watching the videos.

**Table 1:** Twelve internet-based speech-language activities.

Activity name	Content description
Auditory selection of letters	Task of listening to a single sound or word and selecting the letter that matches it from several candidates. Objective: to improve phonetic/phonological recognition and reading of letters
Auditory selection of object drawing	Task of listening to a word and selecting the matching picture from several candidates. Objective: to improve word comprehension
Auditory selection of action drawing	Task of listening to a verb and selecting the matching picture from several candidates. Objective: to improve verb comprehension
Dictation task	Task of listening to a word and writing it down. Objective: to improve dictation
Auditory selection and dictation of numbers	Task of listening to a number and selecting the matching number from several candidates and writing it down. Objective: to improve listening and dictation of numbers
Reading aloud letters	Reading aloud the words on the screen. Objective: to improve reading aloud of letters
Reading aloud current topics	Reading aloud the newspaper article on the screen together. Objective: to improve reading aloud of sentences
Repetition of words and sentences	Task of listening to a word or sentence and repeating it. Objective: to improve repetition
Repetition of nonverbal words	Task of listening to a nonverbal word or sentence and repeating it. Objective: to improve phonological recognition
Matching of object drawing and letters	Task of looking at a picture of an object and selecting the matching letter from several candidates. Objective: to improve word comprehension and reading of letters
Matching of action drawing and letters	Task of looking at a picture of an action and selecting the matching letter from several candidates. Objective: to improve verb/particle/syntax comprehension and reading of letters
Dysarthria training	Task of speaking along with the animation. Objective: to improve articulation

Participants were given an answer/questionnaire booklet and a voice recorder and asked to record themselves during speech-language activity to provide a participation record. Participants wrote the answers in the answer/questionnaire booklet for the two dictation tasks. Questionnaires were completed at the end of each activity session at home to ascertain participants’ subjective impressions of each activity. When undertaking speech-language activities alone at home, it can become increasingly difficult for pwPPA to maintain their motivation, interest, and concentration. Another concerns were that the level of difficulty and the speed of the videos could not be adapted for individuals’ disease progression and that they might not be able to understand the content of the task. Therefore, a questionnaire was administered to determine whether these concerns had arisen in the session. The questionnaire included a seven-point scale with five items, including “Whether or not I would like to do it again,” “Whether or not it was interesting,” “Whether or not I could concentrate on the task,” “Whether or not the task was difficult,” and “Whether the speed of the video was fast or slow.” Considering that pwPPA often have difficulty -answering questionnaires, they were asked to use the 7-point scale by simply circling the appropriate place on the line, which was visually comprehensible (S1 Fig). Activity participation was confirmed according to whether the questionnaire was complete. At the end of 12 weeks (3 courses of activity), participants were asked if they wanted to continue participating in speech-language activity. Those who wished to continue could do so for up to 48 weeks (12 courses) after giving consent again. The group that wished to continue participation after the initial 12-week period was designated the ‘continued’ group, and the group that stopped at that point was designated the ‘discontinued’ group.

### 2.3 Statistical analysis

Because of missing values in the questionnaire items due to non-participation, the results of the questionnaire items for the entire study period were averaged for each activity and applied to the statistics. To conduct a time-series analysis of the questionnaire scores, repeated measures analyses of variance (ANOVAs) were conducted for the average questionnaire scores for each course, followed by Bonferroni’s post hoc test for multiple pairwise comparisons when statistically significant. When Mauchly’s sphericity test was significant, the ANOVA results were adjusted for sphericity using Greenhouse-Geisser correction. The dimensions of the five questionnaire items were reduced to two principal components by principal component analysis. Then the correlation between the principal components and the scores of neuropsychological tests was analyzed using Pearson’s correlation coefficient. The results of participants’ neuropsychological tests were examined with the Shapiro-Wilk test to determine if they were derived from a normal distribution. We included neuropsychological test items that followed a normal distribution, such as MMSE, RCPM, FAB, GDS, and ten sub scores of the Standard Language Test of Aphasia (SLTA), including ‘Following verbal commands,’ ‘Speaking object names,’ ‘Explaining a picture story,’ ‘Sentence repetition,’ ‘Word fluency,’ ‘Following written commands,’ ‘Writing kanji words,’ ‘Narrative writing,’ ‘Dictating kanji words’ and ‘Calculation.’ All statistical analysis was carried out using SPSS version 28.0 (IBM Corp.: Armonk, NY). As our argument about statistical significance in this study comes from an individual null hypothesis, and not a joint null hypothesis *p*<0.05 was considered statistically significant [17].

## 3. Results

### 3.1 Factors related to persistence with speech-language activity on the Internet

**Fig 1:**
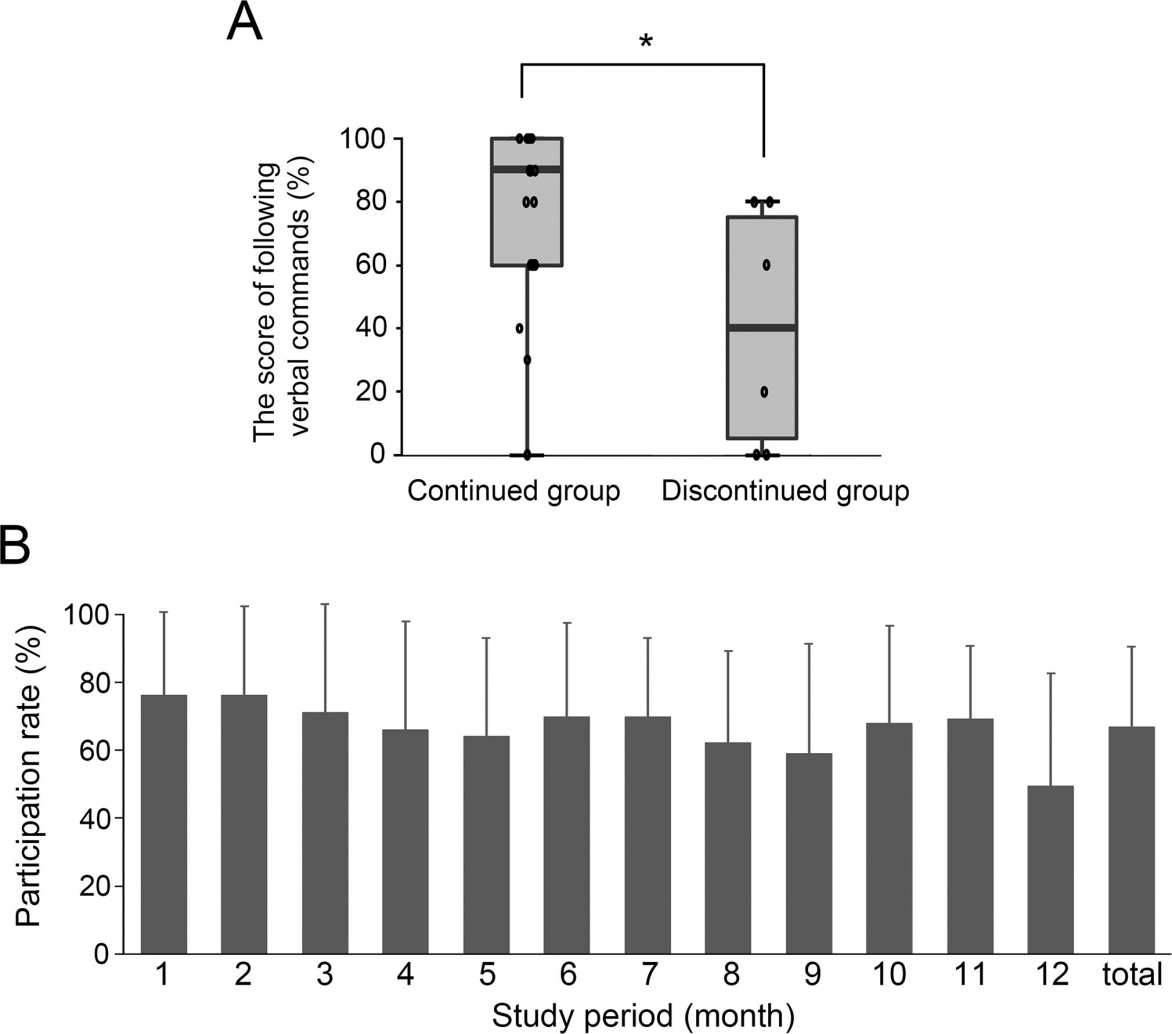
The score of the Standard Language Test of Aphasia (SLTA) subtest related to activity persistence rate and the monthly participation rate. **(**A) The score of ‘Following verbal commands,’ one of the SLTA subtests, was significantly lower in the discontinued group compared with those continuing with the activity. The two groups were classified according to whether they wished to continue the activity at the end of 12 weeks *p < 0.05 (Mann-Whitney U test). (B) The monthly participation rate shows that participation remained relatively high throughout the study.

Participants were asked to confirm in writing whether they would continue their participation at the end of the 12 weeks after three courses of speech-language activity. Of the 23 participants, 17 chose to continue and 6 dropped out, giving a persistence rate of 73.9%. One participant with lvPPA declined to complete the dictation task in advance because of dysgraphia, but the others were able to work on all tasks without avoiding specific ones. We examined the factors related to the three-month persistence rate. There were no statistically significant differences between the continued and discontinued groups in the percentage of PPA sub-diagnostic classifications or in the presence or absence of collaborators to support speech-language activity (Fisher’s direct probability test, *p* = 0.32 and *p* = 0.58, respectively; S1 and S2 Tables). Neuropsychiatric tests revealed no statistically significant differences in MMSE, FAB, RCPM, and GDS scores between the groups (Mann-Whitney U test, *p* = 0.919, *p* = 0.919, *p* = 0.658 and *p* = 0.354, respectively, S2 Fig). Only one of the SLTA subtests–the ‘Following verbal commands’ subtest–was significantly lower in the discontinued group compared with the continued group (Mann-Whitney U test, *p* < 0.05. Fig 1A).

### 3.2 Correlation between the questionnaire content and disease progression

Four participants (of the 17 in the continued group) dropped out because of hospitalization or other reasons. Thus, 13 participants were able to continue the activity for one year. Following the study design, one video was uploaded to the website, and this was changed three times a week. This meant that participants might miss a video, i.e., miss out on participating in some activity sessions. However, the final analysis showed that the monthly participation rate remained relatively high throughout the study period (Fig 1B).

The average questionnaire scores for each task and course are shown in S3 or S4 Figs, respectively. Based on the results shown in S4 Fig, we analyzed whether the subjective impressions of the questionnaire differed among the 12 courses. The perceived speed of the videos differed significantly among all courses (repeated measure ANOVA, p<0.05), indicating that there was some variation in the adjustment of speed in the videos we created, but the other subjective impressions such as motivation, interest, concentration, and video difficulty did not differ significantly among the courses, indicating that these subjective impressions did not change over the time course of their activities.

To make it easier to determine the data characteristics, these subjective impressions were summarized using principal component analysis. Principal component analysis revealed that the five questionnaire items could be reduced to two dimensions: a comprehensive first main component related to motivation, interest and concentration, and a second main component regarding the video speed and difficulty. Eigenvalues exceeded 1.0 for all first principal components, and for the second principal components, except for the ‘Reading aloud current topics’ task. No third principal components with eigenvalues above 1.0 were found for all types of tasks (Table 2). For each activity session, we explored cognitive domains correlated with changes in these principal components to determine the nature of cognitive decline associated with these concerns.

**Table 2:** Eigenvalues and contribution after principal component analysis.

Task name	PC	motivation	interest	Concentration	difficulty	video speed	eigenvalues	contribution rate	cumulative contribution ratio
Auditory selection of letters	1	0.912	0.89	0.848	0.388	-0.201	2.533	50.654	50.654
	2	-0.295	-0.075	0.281	0.686	0.84	1.348	26.967	77.622
Auditory selection of object drawing	1	0.961	0.859	0.752	0.099	0.019	2.237	44.732	44.732
	2	-0.143	-0.454	0.567	0.866	0.792	1.926	38.518	83.25
Auditory selection of action drawing	1	0.901	0.915	0.854	-0.067	0.495	2.627	52.537	52.537
	2	-0.115	-0.353	0.213	0.895	0.615	1.363	27.262	79.799
Dictation task	1	0.923	0.943	0.912	-0.081	-0.064	2.585	51.699	51.699
	2	-0.239	0.219	0.163	0.912	0.941	1.848	36.969	88.668
Auditory selection and dictation of numbers	1	0.832	0.897	0.887	0.367	0.524	2.693	53.86	53.86
	2	-0.481	-0.312	0.055	0.819	0.631	1.402	28.034	81.894
Reading aloud letters	1	0.84	0.792	0.841	0.388	0.53	2.472	49.431	49.431
	2	-0.198	-0.463	-0.077	0.723	0.596	1.137	22.744	72.175
Reading aloud current topics	1	0.874	0.977	0.845	0.689	0.444	3.105	62.09	62.09
	2	-0.217	0.037	-0.326	0.056	0.878	0.928	18.553	80.644
Repetition of words and sentences	1	0.794	0.814	0.85	0.666	0.657	2.89	57.806	57.806
	2	-0.506	-0.478	0.077	0.649	0.446	1.11	22.202	80.008
Repetition of nonverbal words	1	0.89	0.887	0.819	0.853	0.537	3.265	65.3	65.3
	2	-0.338	-0.299	-0.212	0.362	0.804	1.026	20.52	85.82
Matching of object drawing and letters	1	0.918	0.873	0.905	0.166	0.139	2.471	49.418	49.418
	2	-0.137	-0.336	0.215	0.868	0.576	1.263	25.265	74.683
Matching of action drawing and letters	1	0.771	0.807	0.877	0.695	0.486	2.733	54.66	54.66
	2	-0.516	-0.399	-0.13	0.656	0.777	1.477	29.533	84.193
Dysarthria training	1	0.905	0.961	0.919	0.386	0.272	2.809	56.176	56.176
	2	-0.307	-0.137	-0.124	0.77	0.833	1.416	28.313	84.489

Abbreviations: PC, principal component.

The correlations between the first principal component for each task and the scores of cognitive tests and SLTA subtests are shown in Table 3. Sentence repetition and narrative writing were the SLTA subtests that correlated most with the first principal component. In particular, sentence repetition from the SLTA correlated with eight of the twelve task videos but did not correlate with the first principal component of two tasks that involved repetition tasks–repetition of words and sentences, and repetition of nonverbal words (Table 3 and S5A–S5R Fig). The narrative writing of SLTA was correlated with the first principal components of four tasks, were also correlated with the sentence repetition of SLTA. Of these, the first principal component of the ‘dictation task’ task was also correlated with three additional SLTA subtests, including ‘Following verbal commands,’ ‘Speaking object names,’ and ‘Explaining a picture story,’ and with the MMSE. The first principal component of the ‘auditory selection of letters’ task correlated with FAB scores. The RCPM, a nonverbal visuospatial cognitive test, did not correlate with the first principal component of any of the tasks, even though our activity required the visual cognition of watching a video. The first principal component of the ‘dysarthria training’ task–the only motor system rehabilitation activity in this study–was inversely correlated with the GDS.

**Table 3:** Correlation coefficients between the first principal component and neuropsychological tests.

Task name	MMSE	FAB	GDS	Follow verbal commands	Speaking object naming	Explain picture story	Sentence repetition	Word fluency	Narrative writing
Auditory selection of letters	0.172	.486*	0.08	0.364	0.222	0.391	.526*	0.056	.507*
Auditory selection of object drawing	-0.124	-0.064	-0.163	0.073	0.075	0.196	.488*	-0.162	0.216
Auditory selection of action drawing	-0.152	0.165	-0.414	-0.087	-0.183	0.372	0.376	-0.146	0.25
Dictation task	.530*	0.271	0.225	.526*	.550*	.699**	.684**	0.422	.627**
Auditory selection and dictation of numbers	0.182	0.239	-0.317	0.252	0.155	0.499	.543*	0.125	.549*
Reading aloud letters	0.049	0.027	-0.174	0.158	0.203	0.347	.570*	-0.036	0.284
Reading aloud current topics	0.277	0.407	-0.071	0.231	0.1	0.514	.566*	-0.024	.567*
Repetition of words and sentences	-0.107	0.008	-0.396	-0.12	-0.085	0.309	0.4	-0.209	0.152
Repetition of nonverbal words	-0.014	0.336	-0.277	0.125	0.08	0.345	0.441	0.06	0.37
Matching of object drawing and letters	0.199	0.174	-0.006	0.245	0.263	0.383	.602*	0.071	0.363
Matching of action drawing and letters	0.184	0.315	-0.177	0.245	0.111	.545*	.603*	0	0.475
Dysarthria training	-0.158	-0.11	-.622**	-0.249	-0.219	0.206	0.162	-0.214	-0.045

Note that only neuropsychiatric tests that showed statistically significant correlations with the first or second principal components are shown. *p < 0.05, **p < 0.01 (Pearson’s correlation coefficient)

We also analyzed the correlation between the second main component for each task and the scores of cognitive tests and SLTA subtests (S3 Table and S5S and S5T Fig). The second principal component related to video speed and difficulty. In this study, all participants received therapies at the same difficulty level and video speed, irrespective of disease progression. Nevertheless, except for the ‘Repetition of nonverbal words,’ no significant correlation was found between the scores of neuropsychiatric tests and the second main component. The second main component of the ‘Repetition of nonverbal words’ task was inversely correlated with the MMSE and word fluency subtest of the SLTA, suggesting that participants who maintained scores on these tests tended to perceive the video speed and difficulty for this task more strongly.

## 4. Discussion

We studied the feasibility of online video-based speech-language activities at home for outpatients with PPA. In this study, approximately 70% of the pwPPA continued activity for three months and participated in approximately 70.4% of the three weekly sessions (Fig 1B). Even in a group of pwPPA whose MMSE had declined to an average of 18.8 points, our results showed that high participation rates could be achieved.

Dementia is a disease that requires multifaceted support from medical, nursing, and social services because of the decline in activities of daily living caused by cognitive impairments. Since the COVID-19 pandemic, digital transformation has accelerated in dementia care because face-to-face support is often limited. Examples include the application of the Internet of Things (IoT) and artificial intelligence to medical and nursing care, providing remote consultation [18], monitoring [19], and support for daily living [20]. Recent studies have also explored the possibility of speech-language therapy delivered through methods other than the traditional face-to-face sessions. Speech-language therapy delivered remotely by a speech-language pathologist via an Internet videoconferencing system can effectively maintain pwPPA’ language function in the short term [21–23]. The pre-generated Internet video-based tasks employed in this study was not tailormade for individuals. An advantage of this approach is that it provides intensive therapeutic opportunities for many pwPPA with preparation by only a small number of therapists, potentially leading to high efficacy. In contrast, a potential disadvantage was that a therapist does not provide direct support, and pwPPA may not be motivated to persist with the sessions. However, the video-based activity provided in this study demonstrated a high persistence rate, possibly achieved by combining the therapy with support during outpatient visits. The role of outpatient visits in this type of situation requires further study because a protocol in which pwPPA return a booklet, which is also a participation record, to his/her physician at each outpatient visit may have been advantageous for continued participation. ‘Following verbal commands,’ a subtest of the SLTA, was the only cognitive and verbal function that correlated with persistence rate, suggesting this would be a cognitive function that would be helpful in incorporating pwPPA into such an intervention.

The assessment of motivation and setting the difficulty level of the video were considered important for the Internet video-based speech-language activity. Therefore, we used a questionnaire after each task session to examine the indicators of activity adherence to identify pwPPA suited to the activity based on the correlation with impairment patterns in cognitive domains. The questionnaire content was dimensionally reduced to two principal components. The first principal component was an index of motivation, interest, and concentration. Two SLTA subtests, sentence repetition and narrative writing, correlated most with the first principal component. In particular, sentence repetition was considered an important cognitive domain because it correlated with many tasks. Repetition tests are commonly used to identify phonological ability, but grammatical competence may also be required when sentences containing many clauses are used. Studies have shown that examination of sentence repetition can provide information about sentence-level abilities, including morphosyntax, verb morphology and functional words [24, 25]. Narrative writing was another sub-test with many significant correlations. It examines the ability to produce sentences and writing ability. Our results suggest that pwPPA with a marked decline in the ability to comprehend and produce sentence structure have difficulty maintaining motivation, interest, and concentration for video-based speech-language task at home, even if it is a task involving only letters or words. Unexpectedly, there was no significant correlation between the first principal components of the two repetitive tasks, the SLTA subtest and sentence repetition. It should be noted that this result may be non-specific and limited to the participants of this study.

The second principal component was an index of how participants perceived the speed and difficulty of the videos. In the current study, the video playback speed was set to be unchangeable, and it was assumed that pwPPA with more severe aphasia would face more challenges with the speed and difficulty of the videos. However, neither verbal nor nonverbal cognitive domains correlated with the second principal component for most speech therapies. This was probably because the speech rate of our videos was slow enough for many participants, and the familiarity of the words used in this study was higher than average ([15]). Thus, our results indicate that Internet videos–if selected and created appropriately–can be used by a wide range of pwPPA. In contrast, the second principal component was inversely correlated with scores on the MMSE and word fluency task for the repetition task involving nonverbal words. This finding suggests that participants who maintained their scores on these tests tended to perceive the video speed and difficulty for the task more strongly. While nonverbal word repetition primarily involves phonological short-term memory [26], verbal word repetition activates lexical representations in long-term memory, reflecting not just phonological–but also semantic–knowledge, and therefore verbal word repetition is generally more accurate than nonverbal word repetition [27]. This property–that nonverbal word repetition does not require semantic knowledge–may be the reason for the inverse correlation observed here, and nonverbal words requires caution in the use of speech-language activities for people with early PPA.

In the present study, twelve Internet video-based speech-language activity sessions were created and shared. The current study was performed as a feasibility study because the number of participants recruited at our medical institutions was not sufficient to determine the efficacy of the intervention. Because this study used a blanket approach in which all participants participated in all types of Internet video-based activities, there was concern that some types of video tasks would decrease motivation regarding activities in individuals with certain types of aphasia. Consistent with this, our results suggest that there is some Internet video content that is not appropriate for activity motivation in some pwPPA according to impairment profile. Taylor-Rubin et al. showed that motivation was more important in sustaining lexical retrieval than caregiver availability in pwPPA [28]. Therefore, future studies will need to allow pwPPA to choose therapy content that is adapted to their needs. In addition, to observe actual participation rates, this study was conducted with participants who were not forced to watch all the videos. Therefore, there is a statistical concern regarding partial participation dropouts. An additional issue in this study is the lack of feedback from the therapist when participants made errors, failed to complete tasks, or succeeded. To resolve this, future studies would need to record results, such as the percentage of correct responses, and provide opportunities for professional feedback. The results indicate a need for an intervention study to determine the therapeutic effectiveness under conditions that will allow for a stable participation rate in the future.

## 5. Conclusions

Internet video-based speech-language activities conducted at pwPPA’ homes resulted in a high persistence rate and suggests the possibility of incorporating these activities into a new therapy forPPA. The ability to follow oral commands correlated with pwPPA’ willingness to persist with participation. Furthermore, scores of two SLTA subtests–sentence repetition and narrative writing, likely associated with the ability to comprehend and produce sentence structure–were highly correlated with motivation, interest and concentration in several speech-language activities. People with PPA could participate in activities based on the same video irrespective of their disease progression level because the videos were created using carefully selected high-frequency words. Cognitive training using videos at home holds potential for future dementia treatment.

## Supporting information

S1 Fig The seven-point scale to confirm participants’ subjectivity included in the answers and questionnaire booklet. Note that the originals were written in Japanese.
(TIF)

S2 Fig Comparison of the scores of neuropsychological tests between continued and discontinued groups. Notes: Mini-mental state examination, MMSE; Frontal assessment battery, FAB; Raven’s colored progressive matrices, RCPM; Geriatric depression scale, GDS; n.s., not significant (Mann-Whitney U test).
(TIF)

S3 Fig The average self-reported questionnaire scores for each task. We used a seven-point scale with five items, including motivation (A), interest (B), concentration (C), video difficulty (D) and video speed (E) to ascertain participants’ subjective impressions of each activity. Video difficulty and video speed were scored higher when they felt easier and slower, respectively. Mean ± S.D. are shown.
(TIF)

S4 Fig The average self-reported questionnaire scores for each course. We used a seven-point scale with five items, motivation (A), interest (B), concentration (C), video difficulty (D) and video speed (E) to ascertain the participants’ subjective impressions of each course. Mean ± S.D. are shown. When we analyzed whether the questionnaire scores differed among the different courses using the repeated Measures ANOVA, a significant difference is found only in the perceived video speed (E, * *p*<0.05), but not in the subsequent multiple pairwise comparisons. n.s., not significant.
(TIF)

S5 Fig Scatter plots of the first or second principal components of speech-language activities and neuropsychological tests. Note that only those with significant differences are presented in Table 3 and S3 Table.
(TIF)

S1 Table Cross table of continued classification and PPA sub diagnoses. Notes: svPPA, semantic variant PPA; naPPA, non-fluent and/or agrammatic variant PPA; lvPPA, logopenic variant PPA.
(DOCX)

S2 Table Cross table of continued classification and collaborator availability. (DOCX)

S3 Table Correlation coefficients between the second principal component and neuropsychological tests. Note that only neuropsychiatric tests showing statistically significant correlations with the first or second principal components are given. *p < 0.05 (Pearson’s correlation coefficient).
(DOCX)

S1 Appendix Sample video that is shortened version of the ’auditory selection of letters’ task. All S1-S12 videos were conceived by SH and YK and created by SH in accordance with the objectives of the task presented in Table 1. Note that the original video used in this study was approximately 10 minutes long, and English subtitles were not present in the original video.
(MP4)

S2 Appendix Sample video that is shortened version of the ‘auditory selection of object drawing’ task. (MP4)

S3 Appendix Sample video that is shortened version of the ‘auditory selection of action drawing’ task. (MP4)

S4 Appendix Sample video that is shortened version of the ‘dictation task’ task. (MP4)

S5 Appendix Sample video that is shortened version of the ‘auditory selection and dictation of numbers’ task. (MP4)

S6 Appendix Sample video that is shortened version of the ‘reading aloud letters’ task. (MP4)

S7 Appendix Sample video that is shortened version of the ‘reading aloud current topics’ task. (MP4)

S8 Appendix Sample video that is shortened version of the ‘repetition of words and sentences’ task. (MP4)

S9 Appendix Sample video that is shortened version of the ‘repetition of nonverbal words’ task. (MP4)

S10 Appendix Sample video that is shortened version of the ‘matching of object drawing and letters’ task. (MP4)

S11 Appendix Sample video that is shortened version of the ‘matching of action drawing and letters’ task. (MP4)

S12 Appendix Sample video that is shortened version of the ‘dysarthria training’ task. (MP4)
