# Supplementary figures and images for: Feasibility study of Internet video-based speech-language activity for outpatients with primary progressive aphasia
Source: PLoS One. 2023 Jul 13;18(7):e0288468. doi: 10.1371/journal.pone.0288468 (PMC10343066; doi:10.1371/journal.pone.0288468)

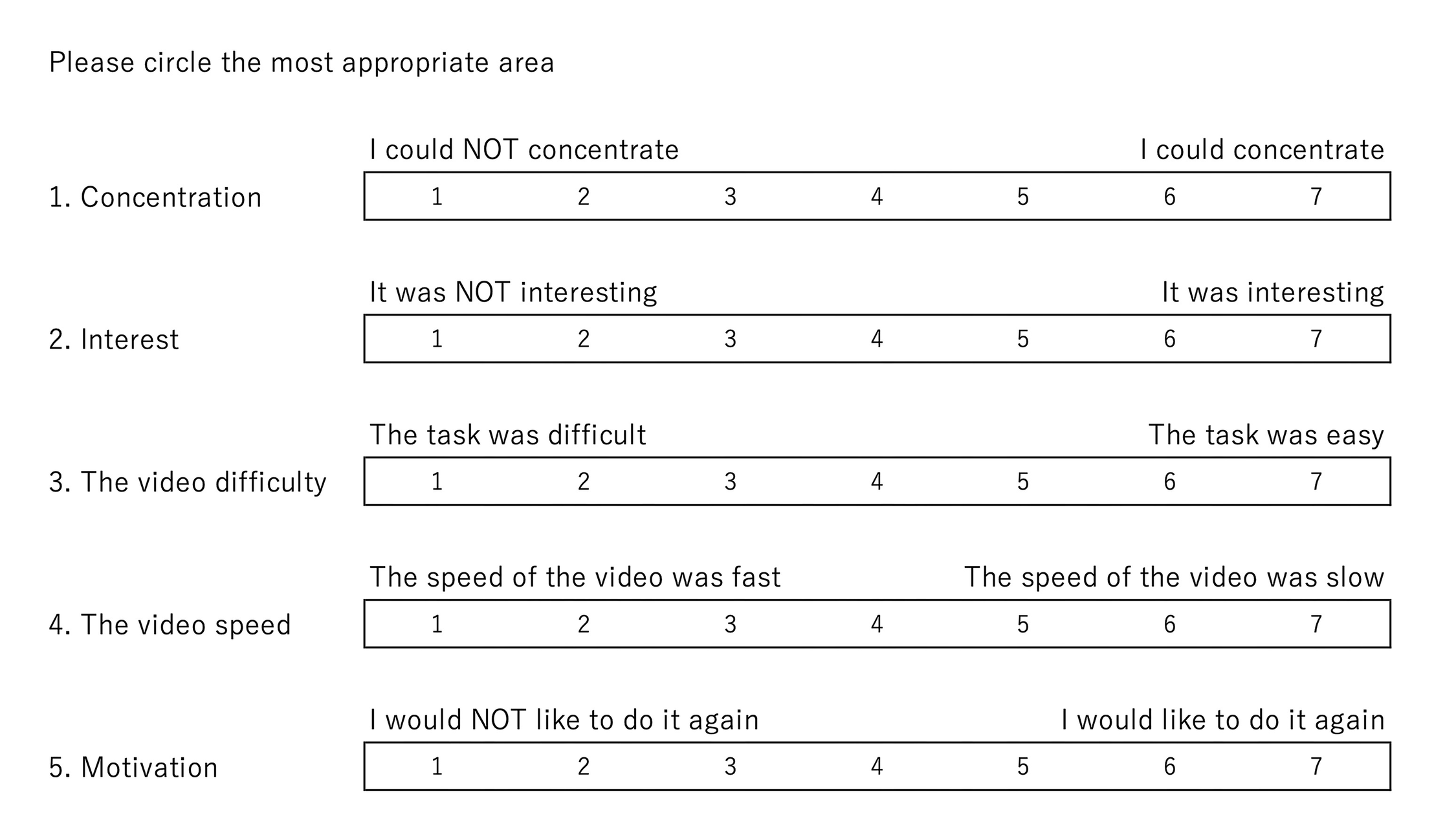

Supplement: S1 Fig — Note that the originals were written in Japanese. (TIF) [file pone.0288468.s001.tif]

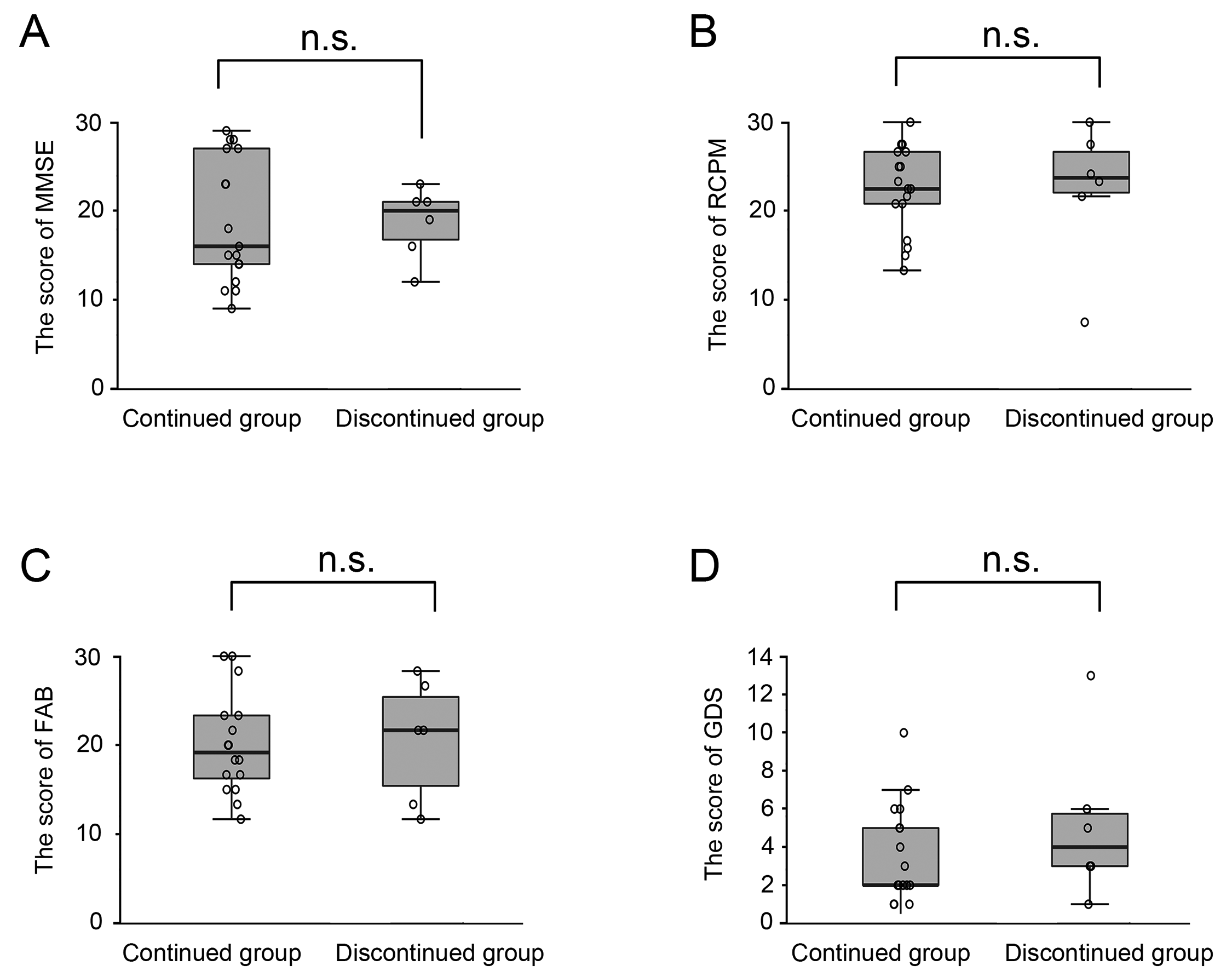

Supplement: S2 Fig — Notes: Mini-mental state examination, MMSE; Frontal assessment battery, FAB; Raven’s colored progressive matrices, RCPM; Geriatric depression scale, GDS; n.s., not significant (Mann-Whitney U test). (TIF) [file pone.0288468.s002.tif]

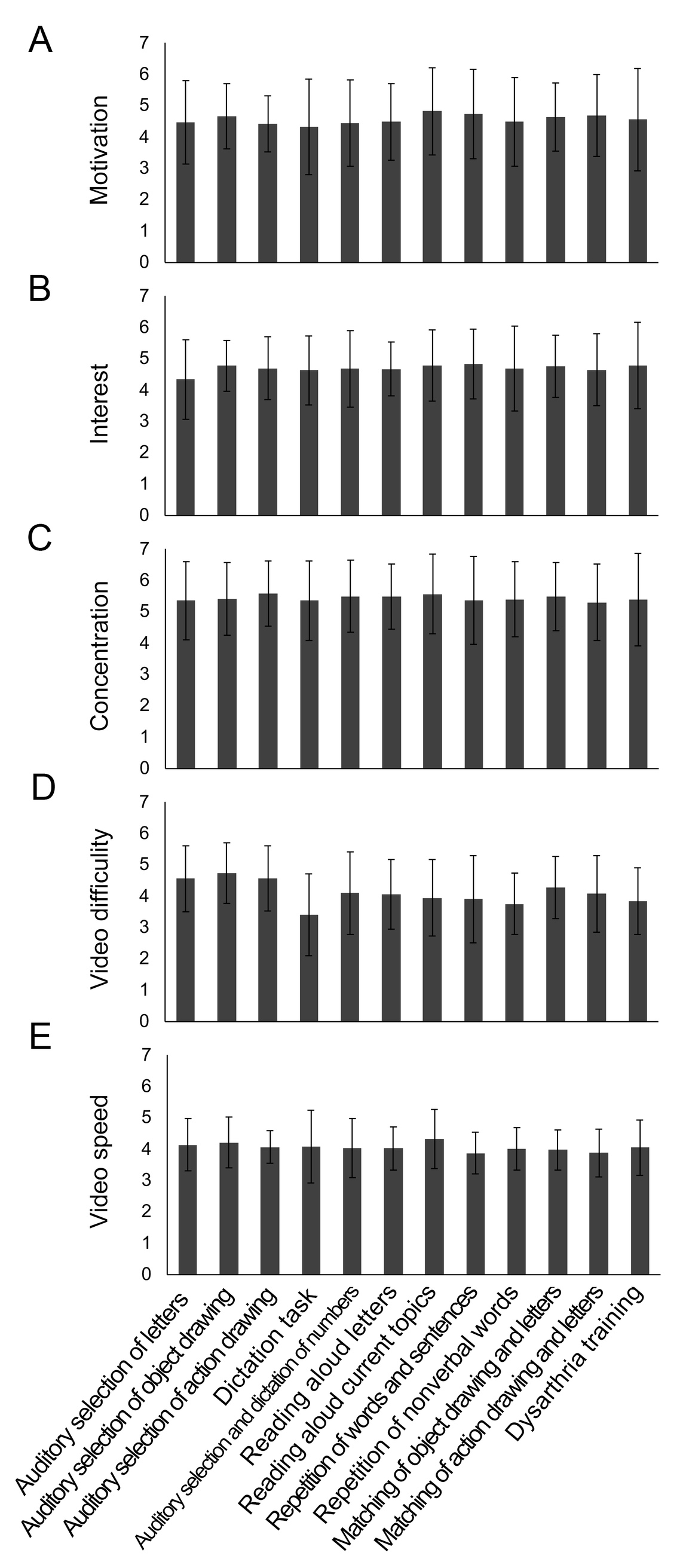

Supplement: S3 Fig — We used a seven-point scale with five items, including motivation (A), interest (B), concentration (C), video difficulty (D) and video speed (E) to ascertain participants’ subjective impressions of each activity. Video difficulty and video speed were scored higher when they felt easier and slower, respectively. Mean ± S.D. are shown. (TIF) [file pone.0288468.s003.tif]

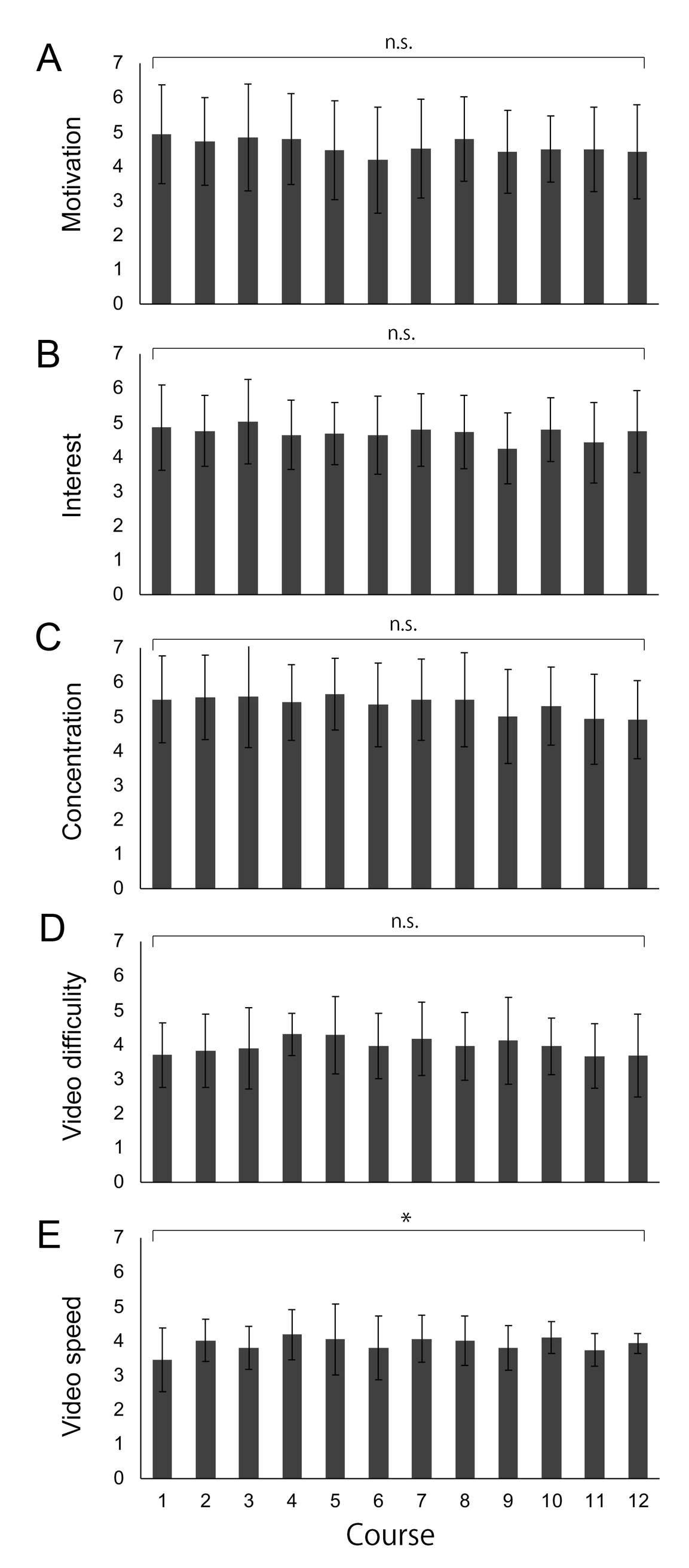

Supplement: S4 Fig — We used a seven-point scale with five items, motivation (A), interest (B), concentration (C), video difficulty (D) and video speed (E) to ascertain the participants’ subjective impressions of each course. Mean ± S.D. are shown. When we analyzed whether the questionnaire scores differed among the different courses using the repeated Measures ANOVA, a significant difference is found only in the perceived video speed (E, * p<0.05), but not in the subsequent multiple pairwise comparisons. n.s., not significant. (TIF) [file pone.0288468.s004.tif]

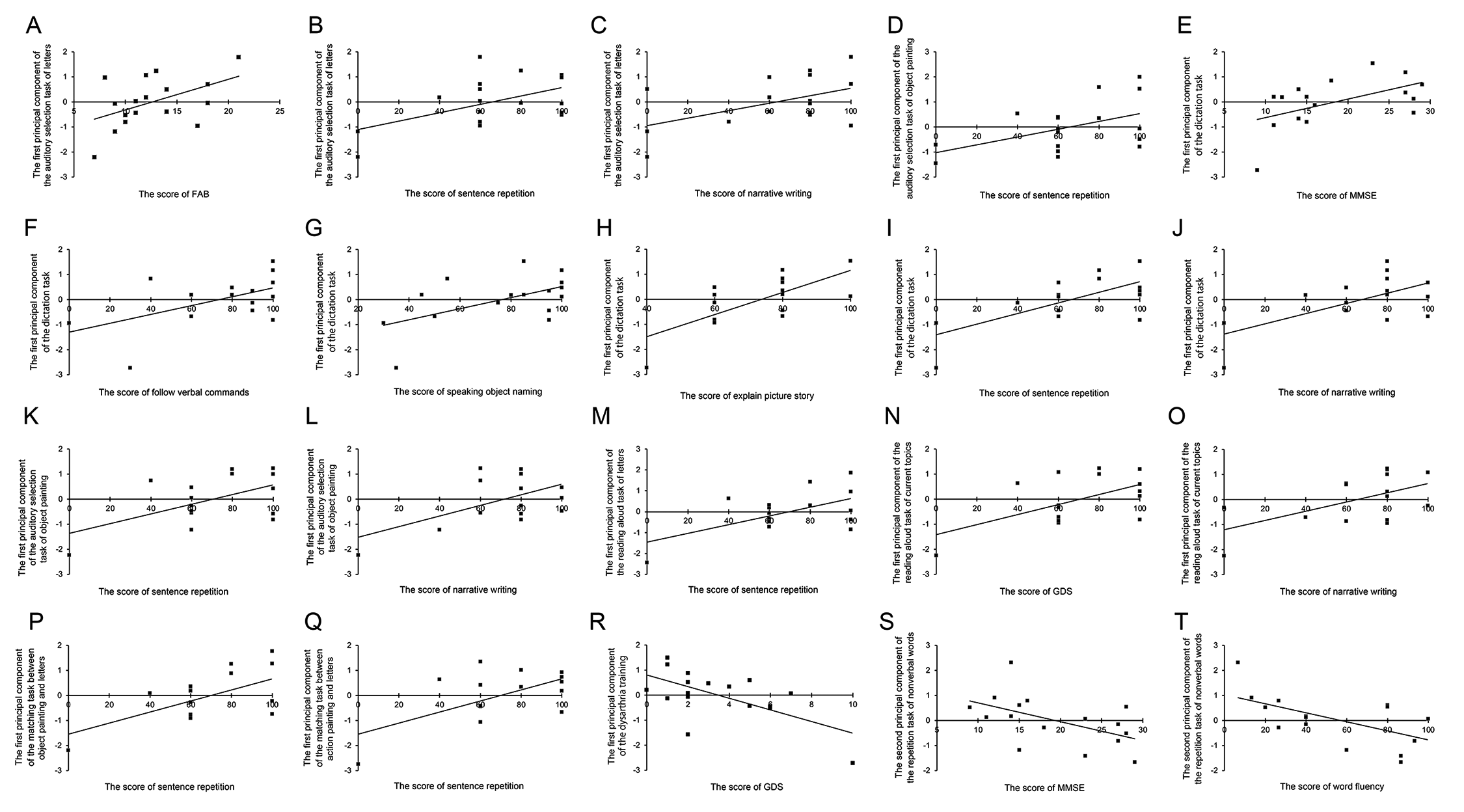

Supplement: S5 Fig — Note that only those with significant differences are presented in Table 3 and S3 Table. (TIF) [file pone.0288468.s005.tif]
