# Supplementary material for: Feasibility study of Internet video-based speech-language activity for outpatients with primary progressive aphasia
Source: PLoS One. 2023 Jul 13;18(7):e0288468. doi: 10.1371/journal.pone.0288468 (PMC10343066; doi:10.1371/journal.pone.0288468)
Supplement: S1 Table — Notes: svPPA, semantic variant PPA; naPPA, non-fluent and/or agrammatic variant PPA; lvPPA, logopenic variant PPA. (DOCX) [file pone.0288468.s006.docx]

**S1 Table. Cross table of continued classification and PPA sub diagnoses.**

|  | lvPPA | naPPA | svPPA | total |
| --- | --- | --- | --- | --- |
| Continued group | 11 | 5 | 1 | 17 |
| Discontinued group | 3 | 1 | 2 | 6 |
| total | 14 | 6 | 3 | 23 |

Notes: svPPA, semantic variant PPA; naPPA, non-fluent and/or agrammatic variant PPA; lvPPA, logopenic variant PPA.
