# Supplementary material for: Feasibility study of Internet video-based speech-language activity for outpatients with primary progressive aphasia
Source: PLoS One. 2023 Jul 13;18(7):e0288468. doi: 10.1371/journal.pone.0288468 (PMC10343066; doi:10.1371/journal.pone.0288468)
Supplement: S2 Table — (DOCX) [file pone.0288468.s007.docx]

**S2 Table. Cross table of continued classification and collaborator availability.**

|  | With collaborator | Without collaborator | total |
| --- | --- | --- | --- |
| Continued group | 14 | 3 | 17 |
| Discontinued group | 4 | 2 | 6 |
| total | 18 | 5 | 23 |
