# Supplementary material for: Feasibility study of Internet video-based speech-language activity for outpatients with primary progressive aphasia
Source: PLoS One. 2023 Jul 13;18(7):e0288468. doi: 10.1371/journal.pone.0288468 (PMC10343066; doi:10.1371/journal.pone.0288468)
Supplement: S3 Table — Note that only neuropsychiatric tests showing statistically significant correlations with the first or second principal components are given. *p < 0.05 (Pearson’s correlation coefficient). (DOCX) [file pone.0288468.s008.docx]

**S3 Table. Correlation coefficients between the second principal component and neuropsychological tests**

| **Therapy name** | **MMSE** | **FAB** | **GDS** | **Follow verbal commands** | **Speaking object naming** | **Explain picture story** | **Sentence repetition** | **Word fluency** | **Narrative writing** |
| --- | --- | --- | --- | --- | --- | --- | --- | --- | --- |
| Auditory selection of letters | 0.116 | 0.209 | -0.406 | -0.039 | -0.219 | 0.205 | -0.112 | -0.012 | -0.14 |
| Auditory selection of object drawing | -0.075 | -0.107 | -0.373 | -0.126 | -0.104 | -0.193 | -0.062 | -0.216 | -0.38 |
| Auditory selection of action drawing | 0.053 | -0.023 | -0.302 | -0.153 | -0.066 | -0.21 | 0.009 | -0.207 | -0.273 |
| Dictation task | -0.056 | -0.058 | -0.251 | -0.215 | -0.291 | 0.311 | -0.119 | -0.176 | 0.039 |
| Auditory selection and dictation of numbers | -0.19 | 0.074 | -0.347 | -0.375 | -0.452 | -0.03 | -0.069 | -0.509 | -0.07 |
| Reading aloud letters | -0.098 | -0.031 | -0.392 | 0 | -0.124 | -0.051 | 0.138 | -0.097 | -0.14 |
| Reading aloud current topics | 0.194 | 0.129 | 0.435 | 0.059 | -0.063 | -0.008 | -0.059 | -0.157 | 0.18 |
| Repetition of words and sentences | -0.044 | 0.001 | -0.057 | 0.093 | 0.031 | -0.214 | 0.159 | -0.197 | -0.042 |
| Repetition of nonverbal words | -.522* | -0.003 | -0.219 | -0.428 | -0.439 | -0.47 | -0.233 | -.617* | -0.24 |
| Matching of object drawing and letters | 0.235 | 0.063 | -0.322 | 0.24 | 0.211 | 0.058 | 0.113 | 0.074 | -0.211 |
| Matching of action drawing and letters | -0.041 | -0.005 | -0.386 | -0.069 | -0.155 | -0.134 | -0.084 | -0.143 | -0.183 |
| Dysarthria training | -0.024 | 0.153 | -0.042 | -0.041 | -0.205 | -0.148 | -0.093 | -0.312 | -0.024 |

Note that only neuropsychiatric tests that showed statistically significant correlations with the first or second principal components are shown. **p* < 0.05 (Pearson’s correlation coefficient)
